# Educational Outcomes in Children and Adolescents With Type 1 Diabetes and Psychiatric Disorders

**DOI:** 10.1001/jamanetworkopen.2023.8135

**Published:** 2023-04-13

**Authors:** Shengxin Liu, Jonas F. Ludvigsson, Paul Lichtenstein, Soffia Gudbjörnsdottir, Mark J. Taylor, Henrik Larsson, Ralf Kuja-Halkola, Agnieszka Butwicka

**Affiliations:** 1 Department of Medical Epidemiology and Biostatistics, Karolinska Institutet, Solna, Sweden; 2 Department of Paediatrics, Örebro University Hospital, Örebro, Sweden; 3 Department of Medicine, Columbia University College of Physicians and Surgeons, New York, New York; 4 Swedish National Diabetes Register, Centre of Registers, Gothenburg, Sweden.; 5 Department of Molecular and Clinical Medicine, Sahlgrenska Academy, University of Gothenburg, Gothenburg, Sweden; 6 School of Medical Sciences, Örebro University, Örebro, Sweden; 7 Child and Adolescent Psychiatry Stockholm, Stockholm Health Care Services, Region Stockholm, Sweden; 8 Department of Child Psychiatry, Medical University of Warsaw, Warsaw, Poland; 9 Department of Biostatistics and Translational Medicine, Medical University of Lodz, Lodz, Poland

## Abstract

**Importance:**

Research shows that children and adolescents with type 1 diabetes (T1D), compared with their peers without diabetes, have a greater risk of psychiatric disorders. However, no study has comprehensively examined whether having psychiatric disorders is associated with educational outcomes in children and adolescents with T1D.

**Objective:**

To investigate educational outcomes in children and adolescents with T1D with and without psychiatric disorders.

**Design, Setting, and Participants:**

This cohort study used data from multiple Swedish registers. The main study cohort included individuals born in Sweden between January 1, 1973, and December 31, 1997, who were followed up from birth through December 31, 2013. Data analyses were conducted from March 1 to June 30, 2022.

**Exposures:**

Type 1 diabetes and psychiatric disorders (including neurodevelopmental disorders, depression, anxiety disorders, eating disorders, bipolar disorder, psychotic disorder, and substance misuse) diagnosed before 16 years of age.

**Main Outcomes and Measures:**

Achieving educational milestones (completing compulsory school [primary and lower secondary education], being eligible to and finishing upper secondary school, and starting and finishing university) and compulsory school performances.

**Results:**

Of 2 454 862 individuals (51.3% male), 13 294 (0.5%; 53.9% male) were diagnosed with T1D (median [IQR] age at diagnosis, 9.5 [6.0-12.5] years), among whom 1012 (7.6%) also had at least 1 psychiatric disorder. Compared with healthy individuals (without T1D and psychiatric disorders), individuals with T1D alone had slightly lower odds of achieving the examined educational milestones. However, those with both T1D and any psychiatric disorder had much lower odds of achieving milestones, including completing compulsory school (odds ratio [OR], 0.17; 95% CI, 0.13-0.21), being eligible for (OR, 0.25; 95% CI, 0.21-0.30) and finishing (OR, 0.19; 95% CI, 0.14-0.26) upper secondary school, and starting (OR, 0.36; 95% CI, 0.29-0.46) and finishing (OR, 0.30; 95% CI, 0.20-0.47) university. They also showed lower grade point averages for compulsory school subjects. These findings remained similar in sibling comparison analyses, suggesting independence from familial confounding.

**Conclusions and Relevance:**

In this cohort study of Swedish-born children and adolescents, those with T1D alone had minor difficulties with their educational outcomes, whereas those with both T1D and psychiatric disorders had universal long-term educational underachievement. These findings highlight the importance of identifying psychiatric disorders in pediatric patients with T1D and the need for targeted educational intervention and support to minimize the education gap between the affected children and their peers.

## Key Points

**Question:**

Do children and adolescents with type 1 diabetes (T1D), with and without psychiatric disorders, have difficulties with educational outcomes?

**Findings:**

In this population-based cohort study of approximately 2.5 million Swedish individuals, those with T1D alone had minor educational underachievement, whereas those with both T1D and psychiatric disorders had universal underachievement in the examined educational outcomes compared with their healthy peers.

**Meaning:**

These findings suggest that target educational supports and adjustments, as well as clinical vigilance, are needed to promote educational outcomes in children and adolescents with T1D and psychiatric disorders.

## Teaser

This cohort study of Swedish children and adolescents with type 1 diabetes examines whether psychiatric disorders are associated with educational outcomes.

## Introduction

Type 1 diabetes (T1D) is one of the most common chronic diseases with onset during childhood.^1^ Children and adolescents with T1D are at risk of psychiatric disorders, such as depression, anxiety, and neurodevelopmental disorders (NDDs) (eg, attention-deficit/hyperactivity disorder [ADHD]).^2,3^ The adverse effects of psychiatric disorders on diabetes management have been well recognized, including less adequate glycemic control, higher risks of diabetic complications, and even premature death.^4,5,6,7,8^ However, the influences may extend to the other life aspects of individuals with T1D. One important aspect is educational attainment, which is a determinant for various life course consequences, such as socioeconomic status and overall adulthood health.^9,10^ Several population-based studies^11,12,13,14,15^ found that T1D alone was associated with increased risks of school absenteeism, lower examination scores, and adulthood unemployment. However, there remains a distinct lack of studies examining educational outcomes in children and adolescents with comorbid T1D and psychiatric disorders.

Moreover, T1D and psychiatric disorders are heritable, with estimated heritability of approximately 50% for T1D^16^ and up to 80% for psychiatric disorders.^17^ Educational attainment is also heritable and depends on familial factors.^18^ Confounding from genetics and familial environmental factors (eg, familial demographic background, socioeconomic status, neighborhood characteristics, and parents’ rearing styles) may exist in the association between T1D, psychiatric disorders, and educational outcomes. Sibling comparison design is a quasi-experimental design used to compare exposed individuals with their unexposed siblings from the same family, controlling for unmeasured confounding from familial factors (ie, genetics and environmental factors shared by siblings).^19^ Thus, a study using a sibling comparison design represents a first step to investigate whether associations are independent of, or attributed to, familial confounding.

The main aim of this study was to examine educational outcomes in children and adolescents with T1D both with and without psychiatric disorders. The secondary aim was to assess whether the associations differ across types of psychiatric disorders. We also aimed to control for familial confounding using a sibling comparison design.

## Methods

### Data Source

For this population-based cohort study using sibling comparison, we obtained data from multiple Swedish nationwide registers. We used the Total Population Register to identify the study cohort and obtain information on migration and vital status^20^; the Multi-Generation Register to identify biological full siblings^20^; Swediabkids database and the National Diabetes Register to identify T1D^21^; the National Inpatient Register,^22^ Clinical Database for Child and Adolescent Mental Health Services,^23^ and Habilitation Register in Stockholm County to obtain psychiatric disorder diagnoses; and the National School Register and the Longitudinal Integrated Database for Health Insurance and Labour Market Studies to retrieve information on educational outcomes.^24^ Supplemental methods are detailed in the eMethods in Supplement 1. Details of used registers are presented in eTable 1 in Supplement 1. Ethical approval was granted by the Regional Ethical Review Board in Stockholm, Sweden. Register-based studies are exempt from informed consent in Sweden.^25^ All data are deidentified. The study followed the Strengthening the Reporting of Observational Studies in Epidemiology (STROBE) reporting guideline for cohort studies.

### Study Population

The initial cohort included 2 607 319 individuals born in Sweden between January 1, 1973, and December 31, 1997, and was followed up until December 31, 2013 (Figure). We excluded individuals diagnosed with chromosomal abnormalities, organic brain disorders, or intellectual disability according to the *International Classification of Diseases, Eighth Revision* (*ICD-8*), *International Classification of Diseases, Ninth Revision* (*ICD-9*), and *International Statistical Classification of Diseases and Related Health Problems, Tenth Revision* (*ICD-10*) codes in eTable 2 in Supplement 1 or who emigrated or died before age 16 years. The final cohort consisted of 2 454 862 individuals, from whom we constructed age-specific subcohorts restricted by birth year and identified families with at least 2 full siblings (Figure).

**Figure.**
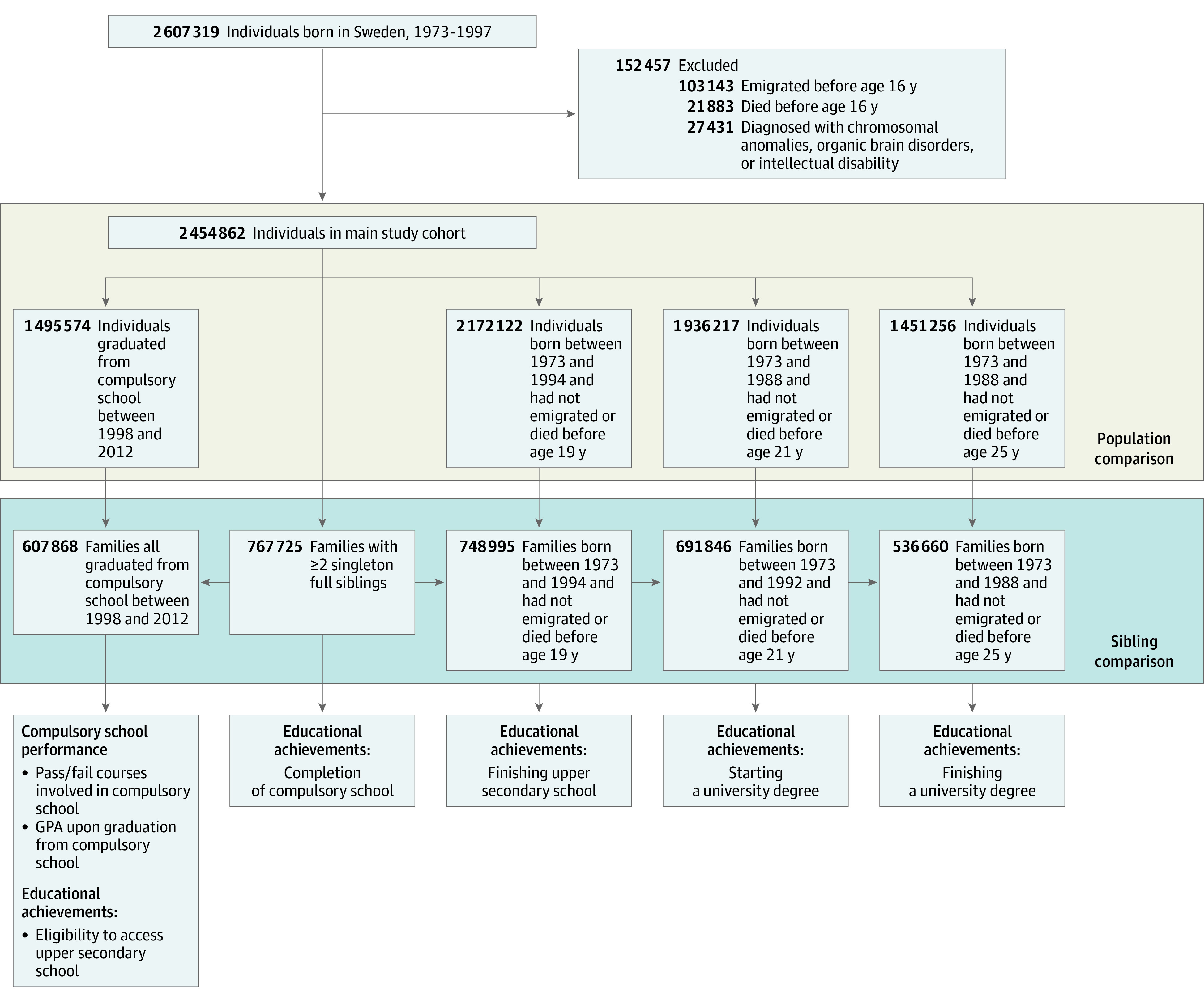
Construction of the Study Cohort GPA indicates grade point average.

### Exposure

Type 1 diabetes was identified from Swediabkids and the National Diabetes Register (diagnostics in eTable 2 in Supplement 1). Psychiatric disorders included NDDs (including autism, ADHD, communication disorders, learning disorders, motor disorders, tic disorders, and unspecified NDDs), depression, anxiety, eating disorders, bipolar disorder, psychotic disorder, and substance misuse. Clinical diagnoses were obtained from the National Inpatient Register, Clinical Database for Child and Adolescent Mental Health Services, and Habilitation Register in Stockholm County using *ICD-8*, *ICD-9*, or *ICD-10* codes (eTable 2 in Supplement 1). In addition to a category of any psychiatric disorder, we made 3 categories based on their prevalence in pediatric diabetes patients^2^ and feature symptoms: (1) NDDs, (2) depression or anxiety, and (3) other psychiatric disorders. We regarded individuals who received a diagnosis before age 16 years as exposed, so the diagnosis was made before the expected age of the first educational milestone.^26^

### Outcome

Educational outcomes included achieving educational milestones and compulsory school performances (eTable 2 in Supplement 1). During the study period, all children in Sweden were required to start compulsory school at approximately 6 years of age, which usually lasts for 9 years and comprises primary and lower secondary education. In the ninth year of compulsory school, students were assessed for eligibility for upper secondary school, which lasted for 3 years. Afterward, students could apply to university to continue tertiary education.

Examined educational milestones included completing compulsory school, being eligible for upper secondary school, finishing upper secondary school, starting university, and finishing university. Postcompulsory milestones were evaluated in age-specific subcohorts so individuals would have sufficient time to achieve each outcome (Figure). The expected age for each milestone was set according to Statistics Sweden.^26^

Compulsory school performances were reflected by the grade point average (GPA) in school subjects and were assessed among individuals who graduated from 1998 to 2012. In 1998 to 2012, 3 core (Swedish, English, and mathematics) and 13 additional subjects were graded during the final academic year. Alphabetical grades were used with corresponding points: Inte-Godkänd (fail, 0 points), Godkänd (pass, 10 points), Väl-Godkänd (pass with distinction, 15 points), and Mycket-Väl-Godkänd (pass with special distinction, 20 points).

### Covariates

We identified other somatic conditions related to educational outcomes,^27^ including asthma, inflammatory bowel disease, epilepsy, and other autoimmune diseases. Information on these conditions diagnosed before age 16 years was obtained from the National Inpatient Register using *ICD-8*, *ICD-9*, and *ICD-10* codes (eTable 2 in Supplement 1). Data on parents’ highest education level before December 31, 2013, were obtained from the Longitudinal Integrated Database for Health Insurance and Labour Market Studies.

### Statistical Analysis

Consistent with previous cohort studies,^28,29,30,31^ we estimated the association of T1D and psychiatric disorders with educational outcomes using logistic regression models for binary outcomes (ie, achieving educational milestones) and linear regression models for continuous outcomes (ie, GPA). For each outcome, we initially modeled T1D and any psychiatric disorder while accounting for their interactions. This approach allowed us to compare individuals with T1D with or without any psychiatric disorder to their healthy peers (ie, without T1D and psychiatric disorder). We then simultaneously modeled T1D and the 3 categories (NDDs, depression or anxiety, and other psychiatric disorders) as independent exposures while accounting for interactions between T1D and the 3 categories. This approach allowed us to compare individuals with T1D with or without each category to their healthy peers while adjusting for the influence of the other categories. Besides the crude model, we fitted an adjusted model to control for sex, birth cohort (6 categories of 5-year intervals), other somatic conditions, and parental highest educational level. The rationale of selected adjusted covariates was detailed in the eFigure in Supplement 1.

We additionally conducted sibling comparison analyses in subcohorts of families with at least 2 full siblings (Figure). By design, this analysis accounts for familial confounding; thus, if associations remained similar in the sibling comparison analyses, one would expect the associations to be independent of familial confounding. We fitted fixed-effect models using conditional logistic and conditional linear regression models in which each family entered as a stratum.^32^ The sibling comparison models were constructed in a similar manner as described above.

For educational milestones, we chose logistic regression models to estimate relative risks because the prevalence of outcomes varied extensively, and logistic regression accommodates any type of outcome prevalence with ease. However, when outcome prevalence is high, inferring relative risk from the odds ratios (ORs) can be less accurate.^33^ Thus, we further estimated risk ratios (RRs) using log-linear regression models to assist finding interpretations. Additionally, we performed sensitivity analyses by restricting analysis to individuals born between 1985 and 1997 to examine whether changes in clinical practices and/or school environments during the later study period affected the associations.

To address our study aims, estimates (ORs and regression coefficients) were calculated and presented for T1D alone, comorbid T1D, and each psychiatric disorder category (namely, T1D and any psychiatric disorder, T1D and NDD, T1D and depression or anxiety, and T1D and other psychiatric disorders). To better understand the role of psychiatric disorders in the observed associations, we also calculated estimates for individuals with psychiatric disorders alone (ie, without T1D).

Data were managed in SAS software, version 9.4 (SAS Institute Inc), and statistical analyses were performed using *drgee*^34^ in R software, version 4.1.2 (R Foundation for Statistical Computing) using a Bonferroni-corrected significance level, determined with 2-sided and unpaired testing, of *P* < .001.^35^ All statistical models used a cluster-robust estimator for SE calculation to control for interfamilial correlation within the data, where clusters were identified via family-specified identifiers.

## Results

Of the 2 454 862 individuals (51.3% male and 48.7% female) in the main cohort, 13 294 (0.5%; 53.9% male and 46.1% female) were diagnosed with T1D at a median (IQR) age of 9.5 (6.0-12.5) years. Among individuals with T1D, 7.6% were clinically diagnosed with at least 1 psychiatric disorder before age 16 years, which is higher than their peers without T1D (3.4%). They were also more likely to have asthma (6.0% vs 3.9%), other autoimmune disorders (9.7% vs 1.3%), and epilepsy (1.4% vs 0.6%) than those without T1D. The highest parental educational level was comparable between individuals with and without T1D (Table 1). Of the total of 767 725 families with at least 2 full siblings, 47 568 families included siblings who were discordant in the exposures (ie, siblings from the same family had different exposure categories) (eTable 3 in Supplement 1).

**Table 1.** Characteristics of the Study Cohort^a^

Characteristic	Type 1 diabetes	
	No (n = 2 441 568)	Yes (n = 13 294)
Sex		
Male	1 251 739 (51.3)	7168 (53.9)
Female	1 189 829 (48.7)	6126 (46.1)
Birth cohort		
1973-1978	575 182 (23.6)	2427 (18.3)
1979-1984	531 536 (21.8)	2470 (18.6)
1985-1990	617 257 (25.3)	3381 (25.4)
1991-1997	717 593 (29.4)	5016 (37.7)
Age at diabetes diagnosis, y		
Mean (SD)	NA	9.1 (4.1)
Median (IQR)	NA	9.5 (6.0-12.5)
Psychiatric disorders		
Any psychiatric disorder	82 872 (3.4)	990 (7.4)
Neurodevelopmental disorders	36 978 (1.5)	361 (2.7)
Depression	12 869 (0.5)	226 (1.7)
Anxiety	22 380 (0.9)	375 (2.8)
Substance misuse	15 583 (0.6)	201 (1.5)
Eating disorders	9501 (0.4)	98 (0.7)
Bipolar and related disorders	924 (0.04)	9 (0.07)
Psychotic disorders	770 (0.03)	8 (0.06)
No. of psychiatric disorders		
1	63 505 (2.6)	759 (5.7)
2	14 137 (0.6)	177 (1.3)
>2	5230 (0.2)	76 (0.6)
Other childhood-onset somatic conditions		
Asthma	96 264 (3.9)	795 (6.0)
Other autoimmune disorders	31 055 (1.3)	1292 (9.7)
Inflammatory bowel disease	4190 (0.2)	32 (0.2)
Epilepsy	14 189 (0.6)	179 (1.4)
Parental highest educational level		
Compulsory education	147 139 (6.0)	701 (5.3)
Upper secondary education	1 142 277 (46.8)	6442 (48.3)
Postsecondary education	1 095 904 (44.9)	5941 (44.7)
Missing	56 248 (2.3)	230 (1.7)

Abbreviation: NA, not applicable.

^a^ Data are presented as number (percentage) of study participants unless otherwise indicated.

Table 2 gives the ORs with 95% CIs of achieving educational milestones in individuals with T1D alone as well as T1D and psychiatric disorders, compared with their healthy peers and full siblings, after multivariable adjustment (estimates for adjusted covariates are presented in eTable 4 in Supplement 1). Compared with their healthy peers, individuals with T1D alone had similar odds of completing compulsory education (OR, 1.09; 95% CI, 0.93-1.28) but statistically significantly slightly lower odds of achieving postcompulsory milestones (ORs, 0.82-0.89).

**Table 2.** Achievement of Educational Milestones in Individuals With T1D With or Without Psychiatric Disorders Compared With Healthy Peers and Full Siblings

Group	Achieved, No. (%)	Odds ratio (95% CI)	
		Adjusted model^a^	Sibling comparison model^b^
**Completion of compulsory school^c^**			
Reference individuals	2 323 274 (98.2)	1 [Reference]	1 [Reference]
T1D alone	12 103 (98.5)	1.09 (0.93-1.28)	0.85 (0.64-1.12)
T1D with any psychiatric disorders	915 (90.4)	0.17 (0.13-0.21)^d^	0.15 (0.09-0.30)^d^
T1D with NDDs	291 (80.6)	0.09 (0.06-0.13)^d^	0.11 (0.04-0.29)^d^
T1D with depression or anxiety	412 (94.0)	0.78 (0.46-1.34)	0.53 (0.16-1.77)
T1D with other psychiatric disorders	212 (93.5)	0.52 (0.30-0.92)	0.10 (0.02-0.46)^d^
**Eligibility for upper secondary school^c^**			
Reference individuals	1 305 636 (90.8)	1 [Reference]	1 [Reference]
T1D alone	7211 (89.6)	0.82 (0.76-0.88)^d^	0.77 (0.66-0.90)^d^
T1D with any psychiatric disorders	564 (73.4)	0.25 (0.21-0.30)^d^	0.35 (0.24-0.51)^d^
T1D with NDDs	140 (60.3)	0.19 (0.14-0.25)^d^	0.21 (0.11-0.42)^d^
T1D with depression or anxiety	289 (76.6)	0.39 (0.30-0.51)^d^	0.39 (0.22-0.69)^d^
T1D with other psychiatric disorders	135 (78.2)	0.47 (0.33-0.68)^d^	1.08 (0.52-2.28)
**Ever finished upper secondary school^c^**			
Reference individuals	1 783 592 (84.0)	1 [Reference]	1 [Reference]
T1D alone	8653 (82.1)	0.84 (0.80-0.88)^d^	0.71 (0.64-0.78)^d^
T1D with any psychiatric disorders	391 (55.5)	0.19 (0.14-0.26)^d^	0.16 (0.08-0.32)^d^
T1D with NDDs	89 (43.8)	0.26 (0.20-0.34)^d^	0.45 (0.28-0.71)^d^
T1D with depression or anxiety	185 (56.3)	0.32 (0.24-0.43)^d^	0.25 (0.13-0.49)^d^
T1D with other psychiatric disorders	117 (47.3)	0.19 (0.14-0.26)^d^	0.16 (0.08-0.32)^d^
**Ever started college or university^c^**			
Reference individuals	830 021 (43.5)	1 [Reference]	1 [Reference]
T1D alone	3612 (39.4)	0.89 (0.85-0.93)^d^	0.79 (0.72-0.86)^d^
T1D with any psychiatric disorders	98 (19.0)	0.36 (0.29-0.46)^d^	0.39 (0.25-0.60)^d^
T1D with NDDs	14 (10.5)	0.26 (0.14-0.47)^d^	0.37 (0.15-0.93)^d^
T1D with depression or anxiety	47 (19.6)	0.42 (0.30-0.59)	0.49 (0.26-0.94)
T1D with other psychiatric disorders	37 (21.1)	0.45 (0.32-0.66)^d^	0.42 (0.21-0.86)^d^
**Ever finished college or university^c^**			
Reference individuals	479 524 (33.4)	1 [Reference]	1 [Reference]
T1D alone	1909 (29.3)	0.86 (0.82-0.92)^d^	0.77 (0.69-0.87)^d^
T1D with any psychiatric disorders	26 (12.5)	0.30 (0.20-0.47)^d^	0.36 (0.14-0.92)^d^
T1D with NDDs	3 (7.1)	NA	NA
T1D with depression or anxiety	9 (11.3)	0.27 (0.13-0.59)	0.63 (0.13-3.11)
T1D with other psychiatric disorders	14 (14.7)	0.43 (0.24-0.76)^d^	0.30 (0.08-1.12)

Abbreviations: NA, not applicable; NDDs, neurodevelopmental disorders; T1D, type 1 diabetes.

^a^ Adjusted for sex, birth cohort, parental highest educational level, and other childhood-onset somatic conditions (any autoimmune disease, asthma, inflammatory bowel disease, or epilepsy).

^b^ Adjusted for sex, birth cohort, and other childhood-onset somatic conditions (any autoimmune disease, asthma, inflammatory bowel disease, or epilepsy) of each individual and full sibling.

^c^ Each outcome was assessed in the corresponding subcohorts shown in the Figure.

^d^ Statistically significant results according to Bonferroni *P* values after correcting for multiple testing.

However, individuals with comorbid T1D and any psychiatric disorder had much lower odds of achieving any of the milestones, including completing compulsory school (OR, 0.17; 95% CI, 0.13-0.21), being eligible for (OR, 0.25; 95% CI, 0.21-0.30) and finishing (OR, 0.19; 95% CI, 0.14-0.26) upper secondary school, and starting (OR, 0.36; 95% CI, 0.29-0.46) or finishing (OR, 0.30; 95% CI, 0.20-0.47) university. All 3 psychiatric disorder categories were associated with lower odds of achieving milestones in individuals with T1D. Those with NDDs had the most profound disadvantages, with considerably lower odds of completing compulsory school (OR, 0.09; 95% CI, 0.06-0.13), being eligible for (OR, 0.19; 95% CI, 0.14-0.25) and finishing (OR, 0.26; 95% CI, 0.20-0.34) upper secondary school, and starting university (OR, 0.26; 95% CI, 0.14-0.47), and only 3 of them has ever finished university. Those with depression, anxiety, or other psychiatric disorders had no statistically significant difference in completing compulsory school (ORs, 0.52-0.78) but statistically significantly lower odds of achieving postcompulsory milestones (ORs, 0.19-0.47). Overall, the estimated ORs of individuals with T1D and psychiatric disorders were comparable to those with psychiatric disorders alone (eTable 5 in Supplement 1).

In the sibling comparison analyses, the associations were largely comparable to those noted in the main cohort. There were a few exceptions where the associations were attenuated, including eligibility for upper secondary school for those with other psychiatric disorders (OR, 1.08; 95% CI, 0.52-2.28), which may be due to low statistical power (suggested by the wide 95% CI).

Estimated RRs supported the observed associations for achieving postcompulsory milestones (eTable 6 in Supplement 1). As we anticipated, the RR for completing compulsory school (RRs, 0.83-0.99) and eligibility for upper secondary school (RRs, 0.69-0.98) were of lesser magnitudes, because these two outcomes were relatively prevalent in the study cohort (all >60%).

Sensitivity analyses in individuals born between 1985 and 1997 yielded similar results as the main analyses but with less precise estimates (eTable 7 in Supplement 1). Finishing university could not be evaluated because most individuals were too young to achieve this milestone.

Compulsory school performances were reflected by the GPAs (a total of 20 points) of school subjects on graduation (Table 3). Compared with their healthy peers, individuals with T1D alone had a slightly lowered GPA for all (β = −0.13; 95% CI, −0.20 to −0.06) and core subjects (β = −0.26; 95% CI, −0.33 to −0.18). However, individuals with T1D and any psychiatric disorder had considerably lower GPAs for all (β = −1.73; 95% CI, −2.00 to −1.46) and core subjects (β = −2.72; 95% CI, −3.02 to −2.42). Those with NDDs had the lowest GPAs, whereas the other 2 categories seemed to have less lowered GPAs. These associations were also comparable to individuals with psychiatric disorders alone (eTable 8 in Supplement 1). Sibling comparison analyses yielded overall similar but less precise estimates. A few associations (eg, GPA for those with other psychiatric disorders) were attenuated to null, more likely because of low statistical power.

**Table 3.** GPA at Graduation From Compulsory School Among Individuals With T1D With or Without Psychiatric Disorders Compared With Healthy Peers and Full Siblings^a^

Group	GPA (a total of 20 points), mean (SD)	Linear regression coefficient, β (95% CI)	
		Adjusted model^b^	Sibling comparison model^c^
**All school courses**			
Reference individuals	13.0 (3.6)	1 [Reference]	1 [Reference]
T1D alone	12.7 (3.6)	−0.13 (−0.20 to −0.06)^d^	−0.19 (−0.28 to −0.09)^d^
T1D with any psychiatric disorder	10.4 (4.4)	−1.73 (−2.00 to −1.46)^d^	−1.12 (−1.50 to −0.75)^d^
T1D with NDDs	8.9 (4.1)	−2.66 (−3.17 to −2.14)^d^	−1.80 (−2.52 to −1.08)^d^
T1D with depression or anxiety	10.7 (4.4)	−1.17 (−1.54 to −0.80)^d^	−0.81 (−1.32 to −0.30)^d^
T1D with other psychiatric disorders	10.8 (4.5)	−0.77 (−1.25 to −0.29)^d^	−0.39 (−1.09 to 0.31)
**Core school courses (Swedish, English, and mathematics)**			
Reference individuals	13.1 (3.5)	1 [Reference]	1 [Reference]
T1D alone	12.9 (3.6)	−0.26 (−0.33 to −0.18)^d^	−0.19 (−0.28 to −0.09)^d^
T1D with any psychiatric disorder	11.5 (3.8)	−2.72 (−3.02 to −2.42)^d^	−1.83 (−2.19 to −1.46)^d^
T1D with NDDs	9.9 (3.6)	−3.31 (−3.84 to −2.78)^d^	−2.24 (−3.07 to −1.78)^d^
T1D with depression or anxiety	11.9 (3.7)	−2.08 (−2.50 to −1.67)^d^	−1.65 (−2.16 to −1.15)^d^
T1D with other psychiatric disorders	12.1 (3.9)	−1.72 (−2.27 to −1.18)^d^	−0.86 (−1.52 to −0.20)

Abbreviations: GPA, grade point average; NDDs, neurodevelopmental disorders; T1D, type 1 diabetes.

^a^ Data from the subcohort of individuals who graduated from compulsory school between the years 1998 and 2012. The GPAs range from 0 to 20.

^b^ Adjusted for sex, birth cohort, parental highest educational level, and other childhood-onset somatic conditions (any autoimmune disease, asthma, inflammatory bowel disease, or epilepsy).

^c^ Adjusted for sex, birth cohort, and other childhood-onset somatic conditions (any autoimmune disease, asthma, inflammatory bowel disease, or epilepsy) of each individual and full sibling.

^d^ Statistically significant results according to Bonferroni *P* values after correcting for multiple testing.

## Discussion

In this population-based cohort study, we found that, compared with their healthy peers, children and adolescents with T1D alone had negligible differences in educational outcomes, whereas those with comorbid T1D and psychiatric disorders fared worse across all educational outcomes. They had more than 50% lower odds of achieving postcompulsory milestones and poorer compulsory school performances. These disadvantages were most likely independent of familial confounding.

Although academic attainment in children and adolescents with T1D has been of research interest, results from prospective studies have been inconclusive. In line with several recent population-based studies,^12,13,14,15^ we observed that those with T1D alone had minor academic underachievement and marginally worse compulsory school performance than their healthy peers.

Conversely, we observed that children and adolescents with comorbid T1D and psychiatric disorders had more worrisome difficulties in achieving education outcomes and school performance. Moreover, these difficulties persisted across specific categories of psychiatric disorders. The NDD group had the most evident adverse educational outcomes, whereas individuals with depression or anxiety or other psychiatric disorders had less but still significant underachievement. These findings align with the few studies that reported associations between psychiatric disorders and educational outcomes in individuals with T1D. One small cross-sectional study^36^ of 39 adolescents with T1D reported that a higher level of depression was associated with lower grades in mathematics, science, and English. A previous study^30^ found that, in Sweden, individuals with both T1D and ADHD experienced more educational difficulties compared with their peers. Nevertheless, studies remain needed to understand whether and how specific psychiatric disorders interfere with educational attainment in patients with T1D.

Several mechanisms can be speculated to explain the observed universal education disadvantages in individuals with comorbid T1D and psychiatric disorders. First, having T1D and psychiatric disorders can interfere with many aspects of the pupils’ school life. For instance, managing diabetes in school can be burdensome for pupils with T1D. They need to devote much attention to continuously monitor their food intake, physical activity, and blood glucose and obtain insulin treatment. These tasks can be even more challenging for those with psychiatric disorders, of which the symptoms can substantially influence the pupils’ self-care abilities. Moreover, our observed estimates for individuals with T1D and psychiatric disorders were similar to estimates for individuals with psychiatric disorders only, suggesting that psychiatric symptoms may directly affect pupils’ learning process and academic performance.^37,38,39^ For example, pupils with NDD often need more time to complete assignments and examinations, and episodes of psychiatric disorders may lead to more school absenteeism. Furthermore, both T1D and behavioral problems have been linked to proneness to school bullying and exclusion in children and adolescents,^40,41,42^ which can be even more psychologically distressing for those with comorbid conditions.

Second, the interplay between T1D and psychiatric disorders on brain functions may be another explanation. Having psychiatric disorders may considerably impair one’s diabetes management,^43,44^ resulting in poor glycemic control, elevated risks of hypoglycemia and ketoacidosis, and frequent diabetes-related hospitalizations. Evidence showed that chronic hyperglycemia, acute hypoglycemia, and ketoacidosis could affect central nervous system development,^45^ causing neurocognitive dysfunctions, such as in attention, working memory, and psychomotor functions. Additionally, blood glucose fluctuations could exacerbate existing psychiatric symptoms by inducing mood changes, irritability, and restlessness,^46^ which can, in turn, affect the patient’s diabetes outcomes. Such a vicious cycle may increase the risk of cognitive dysfunction and, subsequently, educational underachievement.

Sibling comparison analyses further supported that the observed associations were primarily attributable to T1D and psychiatric disorders rather than familial confounding. Although there were slight shifts in the magnitude of the estimates, these shifts were more likely due to low statistical power. However, we could not completely rule out potential contributions from shared familial factors to the observed associations. For instance, pleiotropic genetic effects may exist, accounting for variations in academic achievements and in T1D and psychiatric disorders. In addition, shared environmental factors, such as parents’ well-being, may contribute to the offspring’s educational attainment.

Our study highlights important clinical implications. In support of the current diabetes guidelines,^47,48^ clinical vigilance of psychiatric disorders in pediatric patients with diabetes is warranted. Early detection and timely intervention can minimize the adverse effects of psychiatric disorders not only on diabetes outcomes but also on educational attainment, which can further influence other life course outcomes. Moreover, schools are a unique opportunity to identify mental health needs in pupils with T1D and to provide targeted education adjustments and supports, which help promote school-life balance in pupils with T1D.^49,50^ Furthermore, joint efforts from researchers, pupils and parents, schools, and policy makers are warranted to better understand the needs of these pupils and ways to improve their school life and academic progression.

### Limitations

This study has several limitations. First, despite our large sample size, we could not examine every psychiatric disorder or different psychiatric comorbidities. Second, our data could not capture the up-to-date changes in pediatric diabetes care, such as variations in diabetes regimens and increased screening for psychiatric disorders. This limited our findings’ applicability to contemporary youths and our ability to explore the potential roles of diabetes-related factors (eg, glycemic control) on educational outcomes. Finally, Sweden has free pediatric health care and tuition-free education, with pupils receiving state-provided financial support. Hence, our findings may not be directly generalized and comparable with other countries. The gaps in educational outcomes may be larger in countries where access to school and/or health care is related to socioeconomic status.

## Conclusions

In this cohort study of Swedish-born children and adolescents, we found those with T1D alone had minor differences in their educational outcomes, but those with comorbid T1D and psychiatric disorders had long-term educational underachievement compared with their healthy peers. These disadvantages persisted across types of psychiatric disorders and were largely independent of familial confounding. Our findings highlight the importance of identifying psychiatric disorders in pediatric patients with T1D and the need for intervention and school support to minimize the influence on academic outcomes.

## Notes

Supplement 1. **eMethods.** Graphic Analysis With Directed Acyclic Graphs (DAGs) for a Potential Relationship Between Type 1 Diabetes, Psychiatric Disorders, and Educational Outcomes and Selected Covariates **eFigure.** Proposed Potential Underlying Mechanisms Between Type 1 Diabetes, Psychiatric Disorders and Educational Outcomes in a DAG **eTable 1.** Details on the National Registers Used in the Present Study **eTable 2.** Diagnostic Criteria of Exposure, Exclusion Diagnosis and Covariate, and Definition of Outcomes **eTable 3.** The Number of Families Contains Discordant Sibling Clusters Regarding Both Exposure and Dichotomous Educational Attainments Outcomes **eTable 4.** Estimated Odds Ratios (OR) and 95% Confidence Interval (95%CI) for Control Variables in the Multivariate Adjusted Model for Estimating Association Between Type 1 Diabetes, Any Psychiatric Disorder and Educational Outcomes **eTable 5.** Achieving Educational Milestones in Individuals With Psychiatric Disorders and Without Type 1 Diabetes **eTable 6.** Achieving Educational Milestones in Individuals With Type 1 Diabetes and With or Without Psychiatric Disorders, Compared to Their Healthy Peers and Full-Sibling **eTable 7.** Achieving Educational Milestones in Individuals With Type 1 Diabetes and With or Without Psychiatric Disorders, Compared to Their Healthy Peers and Full-Siblings, Born During 1985-1997 **eTable 8. **Compulsory School Performances in Individuals With Psychiatric Disorders and Without Type 1 Diabetes
eReferences

Supplement 2.
Data Sharing Statement
