# Supplementary material for: Educational Outcomes in Children and Adolescents With Type 1 Diabetes and Psychiatric Disorders
Source: JAMA Netw Open. 2023 Apr 13;6(4):e238135. doi: 10.1001/jamanetworkopen.2023.8135 (PMC10102872; doi:10.1001/jamanetworkopen.2023.8135)
Supplement: Supplement 1. — eMethods. Graphic Analysis With Directed Acyclic Graphs (DAGs) for a Potential Relationship Between Type 1 Diabetes, Psychiatric Disorders, and Educational Outcomes and Selected Covariates eFigure. Proposed Potential Underlying Mechanisms Between Type 1 Diabetes, Psychiatric Disorders and Educational Outcomes in a DAG eTable 1. Details on the National Registers Used in the Present Study eTable 2. Diagnostic Criteria of Exposure, Exclusion Diagnosis and Covariate, and Definition of Outcomes eTable 3. The Number of Families Contains Discordant Sibling Clusters Regarding Both Exposure and Dichotomous Educational Attainments Outcomes eTable 4. Estimated Odds Ratios (OR) and 95% Confidence Interval (95%CI) for Control Variables in the Multivariate Adjusted Model for Estimating Association Between Type 1 Diabetes, Any Psychiatric Disorder and Educational Outcomes eTable 5. Achieving Educational Milestones in Individuals With Psychiatric Disorders and Without Type 1 Diabetes eTable 6. Achieving Educational Milestones in Individuals With Type 1 Diabetes and With or Without Psychiatric Disorders, Compared to Their Healthy Peers and Full-Sibling eTable 7. Achieving Educational Milestones in Individuals With Type 1 Diabetes and With or Without Psychiatric Disorders, Compared to Their Healthy Peers and Full-Siblings, Born During 1985-1997 eTable 8. Compulsory School Performances in Individuals With Psychiatric Disorders and Without Type 1 Diabetes eReferences [file jamanetwopen-e238135-s001.pdf]

Supplementary Online Content

Liu S, Ludvigsson JF, Lichtenstein P, et al. Educational outcomes in children and adolescents with type 1 diabetes and psychiatric disorders. *JAMA Netw Open*. 2023;6(4):e238135. doi:10.1001/jamanetworkopen.2023.8135

eMethods. Graphic Analysis With Directed Acyclic Graphs (DAGs) for a Potential Relationship Between Type 1 Diabetes, Psychiatric Disorders, and Educational Outcomes and Selected Covariates

eFigure. Proposed Potential Underlying Mechanisms Between Type 1 Diabetes, Psychiatric Disorders and Educational Outcomes in a DAG

eTable 1. Details on the National Registers Used in the Present Study

eTable 2. Diagnostic Criteria of Exposure, Exclusion Diagnosis and Covariate, and Definition of Outcomes

eTable 3. The Number of Families Contains Discordant Sibling Clusters Regarding Both Exposure and Dichotomous Educational Attainments Outcomes

eTable 4. Estimated Odds Ratios (OR) and 95% Confidence Interval (95%CI) for Control Variables in the Multivariate Adjusted Model for Estimating Association Between Type 1 Diabetes, Any Psychiatric Disorder and Educational Outcomes

eTable 5. Achieving Educational Milestones in Individuals With Psychiatric Disorders and Without Type 1 Diabetes

eTable 6. Achieving Educational Milestones in Individuals With Type 1 Diabetes and With or Without Psychiatric Disorders, Compared to Their Healthy Peers and Full-Sibling

eTable 7. Achieving Educational Milestones in Individuals With Type 1 Diabetes and With or Without Psychiatric Disorders, Compared to Their Healthy Peers and Full-Siblings, Born During 1985-1997

eTable 8. Compulsory School Performances in Individuals With Psychiatric Disorders and Without Type 1 Diabetes

eReferences

This supplementary material has been provided by the authors to give readers additional information about their work.

© 2023 Liu S et al. *JAMA Network Open.*

eMethods. Graphic Analysis With Directed Acyclic Graphs (DAGs) for a Potential Relationship Between Type 1 Diabetes, Psychiatric Disorders, and Educational Outcomes and Selected Covariates

We used Directed Acyclic Graphs (DAGs) to illustrate the potential mechanisms underlying the association of type 1 diabetes and psychiatric disorders with educational outcomes, and the rationale of the selected covariates. The principles of DAG in epidemiological research have been described in detail elsewhere [7].

As shown in **eFigure 1A**, we present a possible underlying DAG, which begins with type 1 diabetes and psychiatric disorders (exposures). While there is no direct relationship between type 1 diabetes and psychiatric disorders, we assume they share common causes, e.g., familial confounding, which contributes to both type 1 diabetes and psychiatric disorders, as well as to the educational outcomes. Sex and birth cohort are known confounders related to exposures and educational outcomes. Parental highest education level has been linked to offspring's psychiatric disorder and educational achievement. Another set of unmeasured confounding, e.g., genetic susceptibility, could be a common cause of type 1 diabetes and the selected somatic conditions, which have been previously linked to educational outcomes. In the adjusted model, these covariates (grey circles) were controlled. Thus, the path between type 1 diabetes/psychiatric disorders and educational outcomes can be disentangled into the direct effect (red line), or via the unmeasured confounding (blue circle and line) directly or indirectly via psychiatric disorders/type 1 diabetes. In the adjusted sibling comparison model, the unmeasured confounding (blue circle and line) was additionally controlled, and the estimated association would reflect the direct relationship between exposures of type 1 diabetes/psychiatric disorders and educational outcomes. In this scenario, for individuals with type 1 diabetes, having comorbid psychiatric disorders would directly influence their educational outcomes.

Another possible scenario is that, as shown in **eFigure 1B**, there is a direct relationship between type 1 diabetes and psychiatric disorders. In this scenario, having psychiatric disorders can be regarded as a mediator in the relationship between type 1 diabetes and educational outcomes. Thus, after adjusting for the selected covariates (grey circle and line) and the unmeasured confounding (blue circle and line), the association we observed for individuals with comorbid type 1 diabetes and psychiatric disorder can be disentangled into the direct association between type 1 diabetes and educational outcomes, and the indirect association via psychiatric disorders.

The third possible scenario is that, as shown in **eFigure 1C**, psychiatric disorders may be a moderator, indicated by the arrow from psychiatric disorders to the arrow between type 1 diabetes and educational outcomes, which affects the strengths of the existing relationship between type 1 diabetes and education outcomes. Thus, after adjusting the selected covariates and the unmeasured confounding for individuals with both type 1 diabetes and psychiatric disorders, the estimated associations will be the association moderated by psychiatric disorders.

© 2023 Liu S et al. *JAMA Network Open.*

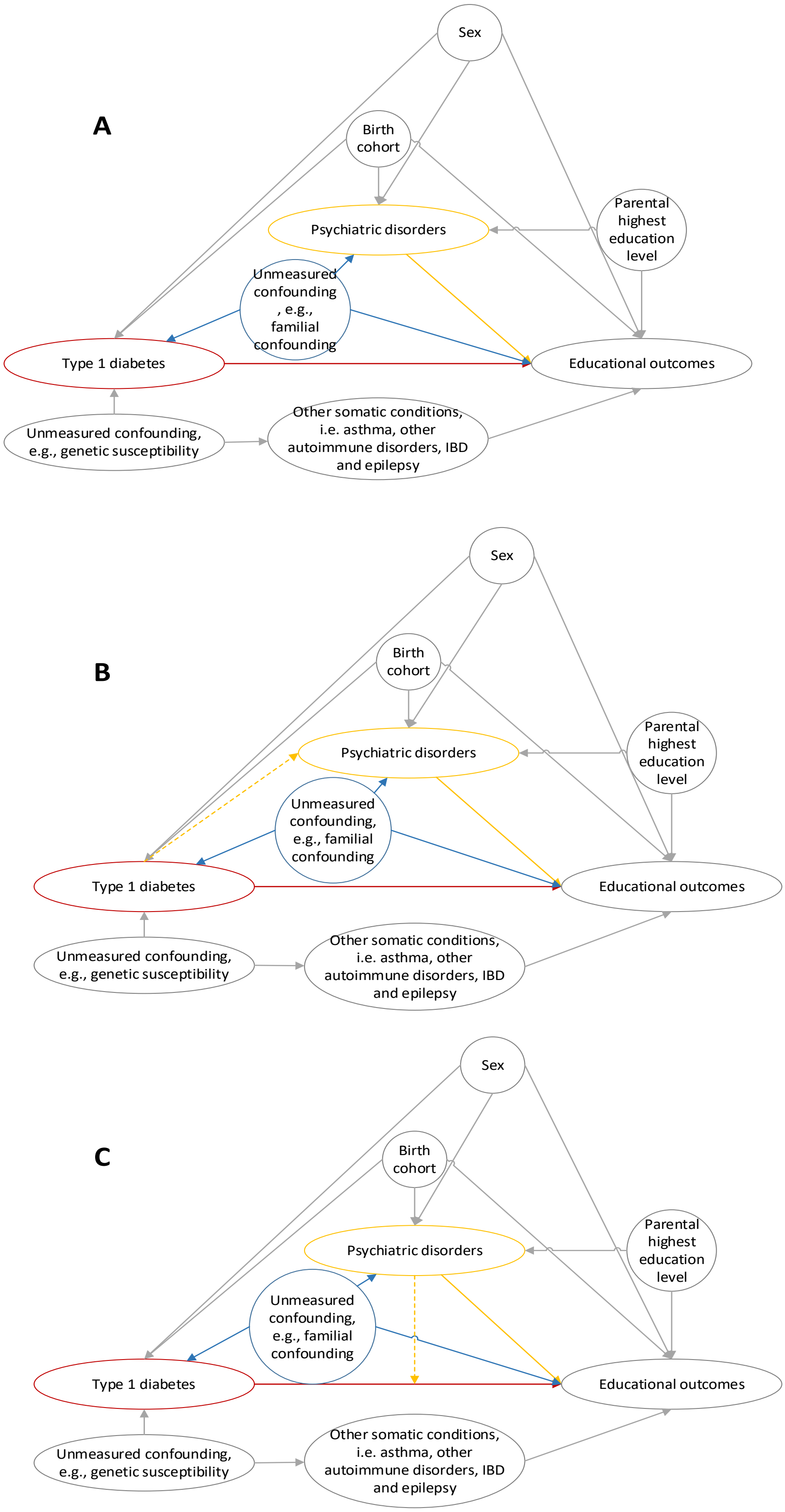

eFigure. Proposed potential underlying mechanisms between type 1 diabetes, psychiatric disorders and educational outcomes in a DAG.

© 2023 Liu S et al. *JAMA Network Open.*

eTable 1 Details on the national registers used in the present study.

Register name, national coverage period	Summary of available information
Total Population Register, 1961-	The Total Population Register covers the complete Swedish population and contains information on their life events, including birth, death, marital status, and migration [1].
Multi-Generation Register, 1932-	The Multi-Generation Register contains information that can link Swedish residents to their biological parents and thus allow identifications of biological relatives [1].
Swedish Diabetes Register, 1996-	The Swedish Diabetes Registry defines type 1 diabetes as receiving insulin treatment and diagnosis before or at age 30, which has been validated in 97% of type 1 diabetes cases [2].
Swediabkids Database, 2000-	The Swediabkids database collected information for more than 95% of Swedish children and adolescents with type 1 diabetes, which contains outpatient records from all pediatric diabetes centers in Sweden [2]. Since 2007 the registry has included data on almost all (98%) children and adolescents with diabetes in Sweden. Diabetes was diagnosed before the age of 18.
National Patient Register, 1964-	The National Patient Register provides information on an inpatient (nationwide coverage since 1987) and outpatient (since 2001) specialist healthcare facilities. Diagnoses were coded using the International Classification of Diseases (ICD 7-10) and were given by a consultant physician at the time of discharge. The validity of many diagnoses has been evaluated and found to be 95-100% [3].
Clinical Database for Child and Adolescent Psychiatry in Stockholm (PASTILL), 2001-	PASTILL provides data from child and adolescent psychiatric inpatient and outpatient care within Region Stockholm. Diagnosis of neurodevelopmental disorders, including attention-deficit/hyperactivity disorder, was proved to have a validity of 96% [4].
Habilitation Register (HAB), 1997-	HAB contains information on the usage of Region Stockholm Habilitation Services due to disability, including intellectual disability, autism spectrum disorder, attention-deficit/hyperactivity disorder, and impairment in motor, vision, or hearing [4].
Medical Birth Register, 1973-	The Swedish Medical Birth Register started in 1973, which includes 99% of all deliveries in Sweden (roughly 100,000 births per year). The register is of high quality. About 90% of all pregnant women have their first antenatal visit in trimester 1 [5].
National School Register, 1985-	The National School Register holds information on individual school performance from all municipal and independent schools from 31st December 1988 [6].
Longitudinal Integration Database for Health Insurance and Labor Studies (LISA), 1990-	LISA integrates annual data on the labor market, education sector, and social sectors for all individuals living in Sweden [6].

© 2023 Liu S et al. *JAMA Network Open.*

eTable 2 Diagnostic criteria of exposure, exclusion diagnosis and covariate, and definition of outcomes.

Diagnostic criteria of exposure, exclusion diagnosis, and covariate diagnosis			
	**ICD-8**<br>1969-1986	**ICD-9**<br>1987-1996	**ICD-10**<br>1997-
**Exposure**			
**Type 1 diabetes**	The diagnosis was obtained from the National Diabetes Register and SWEDIABKIDS database, with ~98% of cases validated.		
**Any psychiatric disorders**	Any code from specific categories of psychiatric disorders.	Any code from specific categories of psychiatric disorders.	Any code from specific categories of psychiatric disorders.
**Neurodevelopmental disorders**			
**Autism spectrum disorders**	-	299	F84
**Attention-deficit/hyperactivity disorder**	-	314	F90
**Communication disorders**	-	315D	F80
**Learning disorders**	-	315A,315B	F81
**Motor disorders**	-	315E	F82
**Tic disorders**	306.2	307C	F95
**Unspecified Neurodevelopmental disorder**	-	315W, 315X	F88, 89
**Depression**	300.4	296B, 300E, 311	F32-F34 (excluding F34.0)<br>F38,F39
**Anxiety disorders**	300.00-300.30, 300.50-300.99, 307	300, 300A-300D, 300F-300X, 308-309	F40-F45, F48
**Substance misuse**	291, 303, 304;	291, 303, 304, 305A, 305X	F10-F19 (excluding F17)
**Eating disorders**	-	307B, 307F	F50
**Bipolar and related disorders**	296	296 (excluding 296B)	F30, F31, F34.0
**Psychotic disorders**	295, 297-299	295, 297, 298	F20-F29

© 2023 Liu S et al. *JAMA Network Open.*

	**Exclusion**		
**Chromosomal abnormality**	759.30-759.50,759.83,310.5, 311.50,312.50,313.50,314.50,315.50	758	Q90-Q99
**Organic brain disorder**	-	-	F00-F09
**Intellectual disability**	310-315	317-319	F70-F79
	**Covariates**		
**Any autoimmune diseases**	Any code from specific categories of autoimmune disease	Any code from specific categories of autoimmune disease	Any code from specific categories of autoimmune disease
**Celiac disease**	269.00,269.99	579A	K90.0
**Hashimoto's thyroiditis**	245.03	245C	E06.3
**Other specified hypothyroidisms**	243.99, 244.00-244.09	243, 244D, 244W, 244X	E03
**Psoriasis**	696.00-696.19,696.98	696A-B,696W	L40
**Rheumatoid arthritis**	712.00-712.50	714A-X	M05, M06, M08
**Thyrotoxicosis**	242.00-242.20	242A-X	E05
**Asthma**	493.00-493.09	493A-X	J45, J46
**Inflammatory bowel disease**	563.10, 563.99, 569.02	555, 556	K51, K50, K52.3
**Epilepsy**	345	345	G40, G41

	**Outcomes**
	**Education milestones**
**Complete compulsory school**	Education in Sweden is mandatory for children between ages 6/7 and 15/16, and normally last for 9 years.
**Being eligible for upper secondary school**	Eligibility to upper secondary school was decided upon graduation from compulsory school, where the pupils have to pass at least the three core subjects.
**Obtaining an upper secondary school**	The upper secondary schools normally last for three years, and most students will be in the national programs. For students not fulfilling the requirements for the national programs, introductory programs are available where students work to satisfy the requirements for the national programs. As previously reported, in Sweden, pupils normally leave secondary education at the age of 19.
**Starting university/college**	The admission to university/college, i.e., starts the tertiary education. This normally follows graduation from upper secondary school, and admission is based mainly on school-leaving grades. To account for the gap year, or taking the Swedish Scholastic Aptitude Test, another way of admission, we decided on age 21 years.
**Finishing university/college**	As previously reported, the vast majority of people graduating from university/college aged about 25 years.

© 2023 Liu S et al. *JAMA Network Open.*

**Compulsory school performances**	
**Grade point average**	Compulsory school subjects include three core subjects (English, Swedish and Mathematics) and thirteen additional subjects (art, biology, chemistry, geography, handcraft, history, home economics, music, physics, religion, society, sports and technology). At the spring semester of 9th year, the teacher graded each subject according to its specific curriculum requirements with alphabetical grades: IG' ('Inte Godkänd', fail), 'G' ('Godkänd', pass), 'VG' ('Väl Godkänd', pass with distinction), and 'MVG' ('Mycket Väl Godkänd', pass with special distinction).<br>The five alphabetical grades are converted into points from 0 to 20, and the grade point average was calculated correspondingly for the three core subjects and all school subjects.

© 2023 Liu S et al. *JAMA Network Open.*

eTable 3 The number of families contains discordant sibling clusters regarding both exposure and dichotomous educational attainments outcomes.

Study cohort	N of families with at least two siblings fulfilled the birth-year cut-off	N of families with at least two siblings that were discordant on the exposure level [a]
**Main cohort**	767,725	55,679
**Sub-cohort: Eligibility to upper secondary school**	607,868	38,492
**Sub-cohort: Finishing upper secondary school**	748,995	38,940
**Sub-cohort: Starting a university degree**	691,846	28,227
**Sub-cohort: Finishing a university degree**	536,660	13,193

[a] ʻDiscordant on the exposure level' means that full-siblings from the same family have different levels of exposure. For example, a family with three siblings, where two with T1D alone and one with T1D and psychiatric disorder, can be regarded as a discordant family. A family with two siblings, where one with T1D and the other without T1D, can also be regarded as a discordant family.

eTable 4 Estimated odds ratios (OR) and 95% confidence interval (95%CI) for control variables in the multivariables adjusted model for estimating the association between type 1 diabetes, any psychiatric disorder, and educational outcomes.

	**Odds Ratios (95% Confidence Interval)** [a]
	**Completion of compulsory school**
**Sex**	
Female vs. male	1.29 (1.27, 1.32)
**Birth cohort**	
1979-1984 vs. 1973-1978	1.39 (1.34, 1.44)
1985-1990 vs. 1973-1978	1.46 (1.41, 1.51)
1991-1997 vs. 1973-1978	0.92 (0.89, 0.94)
**Parental highest education**	
Upper secondary vs. compulsory	1.89 (1.83, 1.96)
Post-secondary vs. compulsory	2.92 (2.81, 3.03)
**Asthma**	0.86 (0.82, 0.90)
**IBD**	1.79 (1.39, 2.29)
**Other autoimmune disorders**	0.69 (0.64, 0.74)
**Epilepsy**	0.13 (0.12, 0.14)
	**Eligibility for upper secondary school** [b]
**Sex**	
Female vs. male	1.37 (1.35, 1.39)
**Birth cohort** [b]	
1985-1990 vs. 1979-1984	0.89 (0.88, 0.91)
1991-1997 vs. 1979-1984	0.90 (0.88, 0.91)
**Parental highest education**	
Upper secondary vs. compulsory	2.12 (2.07, 2.17)
Post-secondary vs. compulsory	6.85 (6.68, 7.02)
**Asthma**	0.92 (0.90, 0.94)
**IBD**	1.13 (0.99, 1.29)
**Other autoimmune disorders**	0.88 (0.84, 0.93)
**Epilepsy**	0.50 (0.47, 0.54)
	**Ever finished upper secondary school**
**Sex**	
Female vs. male	1.57 (1.56, 1.58)
**Birth cohort**	
1979-1984 vs. 1973-1978	1.34 (1.32, 1.35)
1985-1990 vs. 1973-1978	1.35 (1.34, 1.37)
1991-1997 vs. 1973-1978	1.13 (1.12, 1.14)
**Parental highest education**	
Upper secondary vs. compulsory	1.79 (1.77, 1.82)
Post-secondary vs. compulsory	4.73 (4.65, 4.80)
**Asthma**	0.88 (0.86, 0.90)
**IBD**	1.07 (0.97, 1.18)
**Other autoimmune disorders**	0.83 (0.80, 0.86)
**Epilepsy**	0.46 (0.44, 0.48)
	**Ever started college/university**
**Sex**	
Female vs. male	1.96 (1.95, 1.98)
**Birth cohort**	
1979-1984 vs. 1973-1978	0.95 (0.95, 0.96)
1985-1990 vs. 1973-1978	0.60 (0.60, 0.61)

© 2023 Liu S et al. *JAMA Network Open.*

1991-1997 vs. 1973-1978	0.26 (0.25, 0.26)
Parental highest education	
Upper secondary vs. compulsory	1.74 (1.72, 1.77)
Post-secondary vs. compulsory	6.23 (6.13, 6.32)
**Asthma**	0.89 (0.87, 0.91)
**IBD**	1.13 (1.04, 1.23)
**Other autoimmune disorders**	0.90 (0.87, 0.93)
**Epilepsy**	0.62 (0.59, 0.66)
**Ever finished college/university**	
**Sex**	
Female vs. male	2.17 (2.16, 2.19)
**Birth cohort** [c]	
1979-1984 vs. 1973-1978	0.89 (0.89, 0.90)
1985-1990 vs. 1973-1978	0.50 (0.50, 0.51)
**Parental highest education**	
Upper secondary vs. compulsory	1.71 (1.68, 1.74)
Post-secondary vs. compulsory	5.98 (5.87, 6.10)
**Asthma**	0.88 (0.86, 0.91)
**IBD**	1.24 (1.11, 1.39)
**Other autoimmune disorders**	0.83 (0.80, 0.87)
**Epilepsy**	0.62 (0.58, 0.66)

[a] Exposures for the presented model were 'type 1 diabetes', 'any psychiatric disorder' and their interaction

[b] Data from the sub-cohort of individuals who graduated from compulsory school between the years 1998 and 2012. Thus, individuals born 1973-1978 were automatically not included in this sub-cohort, for they will be at youngest 20 years of age in 1998, which exceeded the oldest age of completing compulsory school.

[c] As 'ever finishing college/university' was assessed in the sub-cohort of individuals born before 1988, there was no one from the birth cohort 1991-1997.

© 2023 Liu S et al. *JAMA Network Open.*

eTable 5 Achieving educational milestones in individuals with psychiatric disorders and without type 1 diabetes.

	Adjusted model [b]	Sibling comparison model[c]
	Odds Ratio (95% Confidence Interval)	
**Completion of compulsory school**		
**Any psychiatric disorders**	0.15 (0.14, 0.15)	0.20 (0.18, 0.21)
**NDD**	0.10 (0.09, 0.10)	0.12 (0.11, 0.13)
**Depression or anxiety**	0.71 (0.67, 0.76)	0.49 (0.43, 0.56)
**Other psychiatric disorders**	0.53 (0.49, 0.56)	0.63 (0.55, 0.72)
**Eligibility for upper secondary school [a]**		
**Any psychiatric disorders**	0.15 (0.14, 0.15)	0.20 (0.18, 0.21)
**NDD**	0.22 (0.21, 0.23)	0.25 (0.24, 0.28)
**Depression or anxiety**	0.40 (0.38, 0.41)	0.40 (0.37, 0.43)
**Other psychiatric disorders**	0.45 (0.43, 0.46)	0.58 (0.53, 0.63)
**Ever finished upper secondary school**		
**Any psychiatric disorders**	0.15 (0.14, 0.15)	0.20 (0.18, 0.21)
**NDD**	0.17 (0.17, 0.18)	0.21 (0.20, 0.23)
**Depression or anxiety**	0.30 (0.29, 0.31)	0.37 (0.34, 0.40)
**Other psychiatric disorders**	0.38 (0.36, 0.39)	0.55 (0.51, 0.58)
**Ever started college/university**		
**Any psychiatric disorders**	0.42 (0.41, 0.43)	0.49 (0.47, 0.52)
**NDD**	0.27 (0.26, 0.29)	0.27 (0.24, 0.31)
**Depression or anxiety**	0.48 (0.46, 0.51)	0.55 (0.51, 0.60)
**Other psychiatric disorders**	0.45 (0.42, 0.49)	0.51 (0.44, 0.60)
**Ever finished college/university**		
**Any psychiatric disorders**	0.42 (0.40, 0.44)	0.49 (0.45, 0.53)
**NDD**	0.18 (0.15, 0.21)	0.21 (0.15, 0.27)
**Depression or anxiety**	0.55 (0.53, 0.57)	0.66 (0.61, 0.70)
**Other psychiatric disorders**	0.54 (0.51, 0.57)	0.60 (0.54, 0.68)

[a] Data from the sub-cohort of individuals who graduated from compulsory school between the years 1998 and 2012.
[b] Adjusted for sex, birth cohort, parental highest education level, and other childhood-onset somatic conditions (any autoimmune disease, asthma, inflammatory bowel disease, epilepsy).
[c] Adjusted for sex, birth cohort, and other childhood-onset somatic conditions (any autoimmune disease, asthma, inflammatory bowel disease, epilepsy) of each individual and the full-sibling.

eTable 6 Achieving educational milestones in individuals with type 1 diabetes and with or without psychiatric disorders, compared to their healthy peers and full-sibling.

	Adjusted model [a]	Sibling comparison model [b]
	Risk Ratio (95% Confidence Interval)	
**Completion of compulsory school**		
**Reference individuals**	1 (ref)	1 (ref)
**T1D alone**	0.92 (0.91-0.94)	1
**T1D with any psychiatric disorders**	0.83 (0.79-0.88)	0.93 (0.91-0.95)
**T1D with NDD**	0.99 (0.97-1.01)	0.84 (0.79-0.90)
**T1D with depression or anxiety**	0.98 (0.95-1.00)	0.99 (0.96-1.02)
**T1D with other psychiatric disorders**	0.92 (0.91-0.94)	0.97 (0.94-1.00)
**Eligibility for upper secondary school [a]**		
**Reference individuals**	1 (ref)	1 (ref)
**T1D alone**	0.98 (0.98-0.99)	0.98 (0.97-0.99)
**T1D with any psychiatric disorders**	0.81 (0.78-0.84)	0.86 (0.82-0.91)
**T1D with NDD**	0.69 (0.63-0.77)	0.72 (0.64-0.81)
**T1D with depression or anxiety**	0.88 (0.83-0.92)	0.84 (0.78-0.91)
**T1D with other psychiatric disorders**	0.91 (0.85-0.97)	0.83 (0.75-0.91)
**Ever finished upper secondary school**		
**Reference individuals**	1 (ref)	1 (ref)
**T1D alone**	0.97 (0.97-0.98)	0.96 (0.95-0.97)
**T1D with any psychiatric disorders**	0.66 (0.61-0.70)	0.73 (0.67-0.79)
**T1D with NDD**	0.58 (0.49-0.67)	0.56 (0.22-1.42)
**T1D with depression or anxiety**	0.71 (0.65-0.78)	0.10 (0.04-0.25)
**T1D with other psychiatric disorders**	0.74 (0.67-0.83)	0.68 (0.22-2.11)
**Ever started college/university**		
**Reference individuals**	1 (ref)	1 (ref)
**T1D alone**	0.94 (0.92-0.97)	0.92 (0.89-0.95)
**T1D with any psychiatric disorders**	0.52 (0.44-0.62)	0.59 (0.46-0.75)
**T1D with NDD**	0.35 (0.22-0.58)	0.37 (0.19-0.71)
**T1D with depression or anxiety**	0.58 (0.45-0.74)	0.55 (0.38-0.80)
**T1D with other psychiatric disorders**	0.62 (0.48-0.80)	0.56 (0.40-0.79)
**Ever finished college/university**		
**Reference individuals**	1 (ref)	1 (ref)
**T1D alone**	0.92 (0.89-0.95)	0.90 (0.86-0.95)
**T1D with any psychiatric disorders**	0.42 (0.3-0.60)	0.57 (0.32-1.01)
**T1D with NDD**	-	-
**T1D with depression or anxiety**	0.39 (0.21-0.74)	0.29 (0.09-0.88)
**T1D with other psychiatric disorders**	0.56 (0.36-0.86)	0.37 (0.19-0.71)

Abbreviations: T1D, type 1 diabetes; NDD, neurodevelopmental disorders

[a] Data from the sub-cohort of individuals who graduated from compulsory school between the years 1998 and 2012.

[b] Adjusted for sex, birth cohort, parental highest education level, and other childhood-onset somatic conditions (any autoimmune disease, asthma, inflammatory bowel disease, epilepsy).

[c] Adjusted for sex, birth cohort, and other childhood-onset somatic conditions (any autoimmune disease, asthma, inflammatory bowel disease, epilepsy) of each individual and the full-sibling.

[d] Only three individuals have achieved this milestone

© 2023 Liu S et al. *JAMA Network Open.*

eTable 7 Achieving educational milestones in individuals with type 1 diabetes and with or without psychiatric disorders, compared to their healthy peers and full-siblings, born during 1985-1997.

	Adjusted model [a]	Sibling comparison model [b]
	Odds Ratio (95% Confidence Interval)	
**Completion of compulsory school**		
**Reference individuals**	1 (ref)	1 (ref)
**T1D alone**	0.92 (0.91-0.94)	1
**T1D with any psychiatric disorders**	1.07 (0.88, 1.30)	0.88 (0.61, 1.28)
**T1D with NDD**	0.16 (0.13, 0.21)	0.16 (0.09, 0.31)
**T1D with depression or anxiety**	0.09 (0.07, 0.13)	0.10 (0.04, 0.27)
**T1D with other psychiatric disorders**	0.85 (0.48, 1.48)	0.55 (0.14, 2.12)
**Eligibility for upper secondary school [c]**		
**Reference individuals**	1 (ref)	1 (ref)
**T1D alone**	0.83 (0.77, 0.90)	0.82 (0.69, 0.97)
**T1D with any psychiatric disorders**	0.25 (0.21, 0.30)	0.30 (0.20, 0.45)
**T1D with NDD**	0.19 (0.14, 0.25)	0.24 (0.12, 0.47)
**T1D with depression or anxiety**	0.39 (0.30, 0.51)	0.29 (0.16, 0.54)
**T1D with other psychiatric disorders**	0.48 (0.33, 0.70)	1.13 (0.49, 2.61)
**Ever finished upper secondary school**		
**Reference individuals**	1 (ref)	1 (ref)
**T1D alone**	0.82 (0.77, 0.88)	0.70 (0.60, 0.82)
**T1D with any psychiatric disorders**	0.21 (0.18, 0.25)	0.28 (0.19, 0.41)
**T1D with NDD**	0.19 (0.14, 0.27)	0.16 (0.08, 0.34)
**T1D with depression or anxiety**	0.27 (0.21, 0.36)	0.43 (0.25, 0.74)
**T1D with other psychiatric disorders**	0.33 (0.24, 0.46)	0.36 (0.17, 0.75)
**Ever started college/university**		
**Reference individuals**	1 (ref)	1 (ref)
**T1D alone**	0.95 (0.89, 1.02)	0.89 (0.76, 1.04)
**T1D with any psychiatric disorders**	0.39 (0.30, 0.51)	0.47 (0.27, 0.83)
**T1D with NDD**	0.28 (0.15, 0.53)	0.58 (0.21, 1.59)
**T1D with depression or anxiety**	0.47 (0.32, 0.68)	0.56 (0.26, 1.20)
**T1D with other psychiatric disorders**	0.46 (0.30, 0.71)	0.49 (0.17, 1.46)
**Ever finished college/university [d]**		
**Reference individuals**	1 (ref)	1 (ref)
**T1D alone**	-	-
**T1D with any psychiatric disorders**	-	-
**T1D with NDD**	-	-
**T1D with depression or anxiety**	-	-
**T1D with other psychiatric disorders**	-	-

Abbreviations: T1D, type 1 diabetes; NDD, neurodevelopmental disorders

[a] Adjusted for sex, birth cohort, parental highest education level, and other childhood-onset somatic conditions (any autoimmune disease, asthma, inflammatory bowel disease, epilepsy).

[b] Adjusted for sex, birth cohort, and other childhood-onset somatic conditions (any autoimmune disease, asthma, inflammatory bowel disease, epilepsy) of each individual and the full-sibling.

[c] Data from the sub-cohort of individuals who graduated from compulsory school between the years 1998 and 2012.

[d] The majority these individuals (born 1985-1997) does not ful-fill the birth-year cut-off (born 1973-1988) for assessing this educational milestone.

© 2023 Liu S et al. *JAMA Network Open.*

eTable 8 Compulsory school performances in individuals with psychiatric disorders and without type 1 diabetes [a].

	Adjusted model [a]	Sibling comparison model [b]
	Linear regression coefficient (β) (95% Confidence Interval)	
**GPA, all school subjects**		
**Reference individuals**	Reference	Reference
**Any psychiatric disorders**	-1.47 (-1.50, -1.44)	-1.04 (-1.08, -1.00)
**NDD**	-2.06 (-2.11, -2.01)	-1.65 (-1.72, -1.57)
**Depression or anxiety**	-0.87 (-0.92, -0.82)	-0.75 (-0.83, -0.68)
**Other psychiatric disorders**	-0.75 (-0.81, -0.70)	-0.38 (-0.45, -0.31)
**GPA, core school subjects (Swedish, English, and Mathematics)**		
**Reference individuals**	Reference	Reference
**Any psychiatric disorders**	-2.53 (-2.56, -2.49)	-1.74 (-1.78, -1.69)
**NDD**	-2.85 (-2.90, -2.80)	-2.13 (-2.20, -2.06)
**Depression or anxiety**	-2.06 (-2.12, -2.00)	-1.66 (-1.73, -1.58)
**Other psychiatric disorders**	-1.50 (-1.56, -1.44)	-0.81 (-0.88, -0.74)

[a] Data from the sub-cohort of individuals who graduated from compulsory school between the years 1998 and 2012. Grade point average ranges from 0 to 20.

[b] Adjusted for sex, birth cohort, parental highest education level, and other childhood-onset somatic conditions (any autoimmune disease, asthma, inflammatory bowel disease, epilepsy).

[c] Adjusted for sex, birth cohort, and other childhood-onset somatic conditions (any autoimmune disease, asthma, inflammatory bowel disease, epilepsy) of each individual and the full-sibling.

eReferences

1. Ludvigsson JF, Almqvist C, Bonamy A-KE, et al. Registers of the Swedish total population and their use in medical research. *European journal of epidemiology*. 2016;31(2):125-136.
2. Lind M, Pivodic A, Svensson A-M, Ólafsdóttir AF, Wedel H, Ludvigsson J. HbA1c level as a risk factor for retinopathy and nephropathy in children and adults with type 1 diabetes: Swedish population based cohort study. *BMJ*. 2019;366
3. Ludvigsson JF, Andersson E, Ekbom A, et al. External review and validation of the Swedish national inpatient register. *BMC public health*. 2011;11(1):450.
4. Idring S, Rai D, Dal H, et al. Autism spectrum disorders in the Stockholm Youth Cohort: design, prevalence and validity. *PloS one*. 2012;7(7):e41280.
5. Axelsson O. The Swedish medical birth register. *Acta Obstet Gynecol Scand*. Jun 2003;82(6):491-2. doi:10.1034/j.1600-0412.2003.00172.x
6. Ludvigsson JF, Svedberg P, Olén O, Bruze G, Neovius M. The longitudinal integrated database for health insurance and labour market studies (LISA) and its use in medical research. *European journal of epidemiology*. 2019;34(4):423-437.
7. Greenland S, Pearl J, Robins JM. Causal diagrams for epidemiologic research. *Epidemiology*. 1999:37-48.

© 2023 Liu S et al. *JAMA Network Open.*
