# Supplementary material for: Educational Outcomes in Children and Adolescents With Type 1 Diabetes and Psychiatric Disorders
Source: JAMA Netw Open. 2023 Apr 13;6(4):e238135. doi: 10.1001/jamanetworkopen.2023.8135 (PMC10102872; doi:10.1001/jamanetworkopen.2023.8135)
Supplement: Supplement 2. — Data Sharing Statement [file jamanetwopen-e238135-s002.pdf]

Data Sharing Statement

Liu. Educational Outcomes in Children and Adolescents With Type 1 Diabetes and Psychiatric Disorders. *JAMA Netw Open*. Published April 13, 2023.
doi:10.1001/jamanetworkopen.2023.8135

Data
Data available: No
